# Evodiamine Attenuates PDGF-BB-Induced Migration of Rat Vascular Smooth Muscle Cells through Activating PPARγ

**DOI:** 10.3390/ijms161226093

**Published:** 2015-11-26

**Authors:** Xie Ge, Siyu Chen, Mei Liu, Tingming Liang, Chang Liu

**Affiliations:** Jiangsu Key Laboratory for Molecular and Medical Biotechnology and College of Life Sciences, Nanjing Normal University, Nanjing 210023, China; gexie85@163.com (X.G.); chensiyujimmy@163.com (S.C.); liumei@njnu.edu.cn (M.L.); tmliang@njnu.edu.cn (T.L.)

**Keywords:** evodiamine, vascular smooth muscle cells, migration, peroxisome proliferator-activated receptor γ

## Abstract

The uncontrolled migration of vascular smooth muscle cells (VSMCs) into the intima is a critical process in the development of atherosclerosis. Evodiamine, an indole alkaloid extracted from the Chinese medicine evodia, has been shown to inhibit tumor cell invasion and protect the cardiovascular system, but its effects on VSMCs remain unknown. In the present study, we investigated the inhibitory effects of evodiamine on the platelet-derived growth factor-BB (PDGF-BB)-induced VSMC migration using wound healing and transwell assays, and assessed its role in decreasing the protein levels of matrix metalloproteinases and cell adhesion molecules. More importantly, we found that evodiamine activated the expression and nuclear translocation of peroxisome proliferator-activated receptor γ (PPARγ). Inhibition of PPARγ activity by using its antagonist T0070907 and its specific siRNA oligonucleotides significantly attenuated the inhibitory effects of evodiamine on VSMC migration. Taken together, our results indicate a promising anti-atherogenic effect of evodiamine through attenuation of VSMC migration by activating PPARγ.

## 1. Introduction

The migration of vascular smooth muscle cells (VSMCs) occurs under both physiological (e.g., vascular development) and pathological (e.g., atherogenesis responsive to vascular injury) circumstances. During vascular development, chemoattractants such as platelet-derived growth factor-BB (PDGF-BB) stimulate migration of pericytes or other smooth muscle precursors, leading to the correct formation of vessel wall structure [1]. In contrast, accelerated VSMC migration contributes importantly to neo-intimal hyperplasia observed in atherosclerosis and restenosis. Usually, balloon catheterization or stent placement triggers the endothelial injury and subsequent abberant VSMC migration, and then the cells migrate from the adventitial and the medial layers to the intimal layer of the vessel wall. Followed by their abnormal migration, VSMC proliferation starts and causes arterial thickening [2].

Migration of VSMCs is a very complicated process and is finely regulated by multiple factors. One of the most important mechanisms involves the crosstalk between inflammation and oxidative stress. Inflammatory cytokines, which are involved in the development and progression of inflammation, function robustly in the regulation of VSMC migration [3]. In addition, chronic and mild inflammation leads to the increased generation of reactive oxygen species (ROS), which induces VSMC migration through activating a series of signal molecules, including protein tyrosine phosphatases/kinases, extracellular signal-regulated kinase (ERK)/mitogen-activated protein kinase (MAPK), and transcription factors [4]. Once VSMC migration is initiated, the expression and secretion of matrix metalloproteinases (MMPs) are upregulated, which facilitates the degradation of vascular basement membrane and extracellular matrix (ECM) proteins [3].

Given the importance of VSMC migration in the pathogenesis of cardiovascular diseases, inhibiting this pathological process may be beneficial for the prevention or alleviation of angiogenesis and other kinds of vascular injury. In fact, retardment of excess smooth muscle cell migration through targeted disruption of MMP-9 leads to reduced intimal hyperplasia and improves arterial remodeling [5]. In addition, the inhibition of VSMC migration by rapamycin and paclitaxel is a contributing factor in their beneficial effects of incorporation into vascular stents [1]. Similarly, as the inhibitors of VSMC migration, peroxisome proliferator-activated receptor γ (PPARγ) activators can also be used for the treatment of cardiovascular diseases [6,7,8]. For example, troglitazone, as a PPARγ agonist, inhibits the activation of ERK, the degradation of p27 (Kip1), the phosphorylation of retinoblastoma protein, and the progression of cell cycle from G1 to S, and thus collectively leads to the reduction of VSMC migration [9].

**Figure 1:**
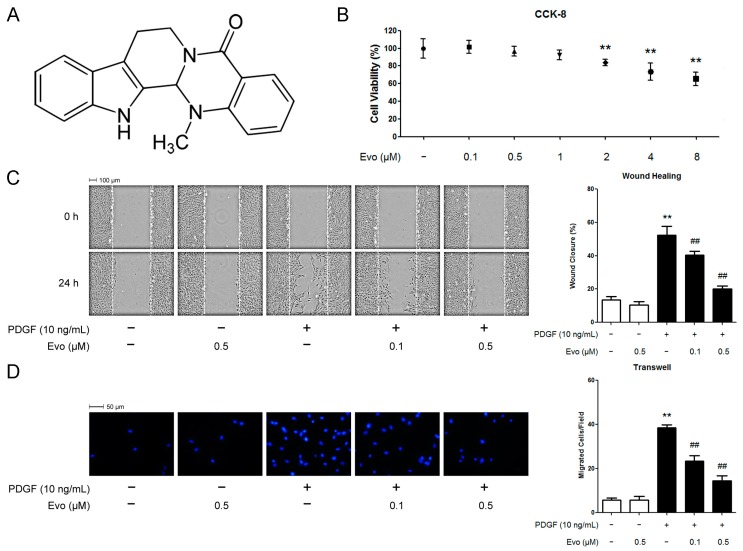
Evodiamine cytotoxicity test and its effect on platelet-derived growth factor (PDGF) -BB-induced vascular smooth muscle cell (VSMC) migration. (**A**) Chemical structure of evodiamine; (**B**) Cytotoxicity test using evodiamine. VSMCs were cultured in medium containing 10% fetal bovine serum, and treated with evodiamine at the indicated concentrations for 30 h. Cell viability was measured using the Cell Counting Kit-8 (CCK-8) assay. Results are expressed as cell viability relative to untreated controls; (**C**,**D**) Evodiamine inhibits PDGF-BB-induced VSMC migration; (**C**) Serum-starved cells were pretreated with evodiamine for 6 h before the stimulation of PDGF-BB for 24 h. Thereafter, cell migration was measured by using a wound healing assay; (**D**) Cells seeded into the inner chamber in serum-free medium were exposed to evodiamine with PDGF-BB for 6 h. Cell migration was thereafter measured by using a Boyden chamber assay. Blue color indicates cell nuclei stained with 4′,6-diamidino-2-phenylindole (DAPI). Evo, evodiamine. Data are represented as mean values ± SD of three independently prepared samples each with five measurements. ** *p* < 0.01 compared with the control group; ^##^ *p* < 0.01 compared with the PDGF-BB-stimulated group.

However, the compounds mentioned above have serious side-effects. Rapamycin (an inhibitor of mammalian target of rapamycin (mTOR)) and paclitaxel (a tubulin-binding agent) encounter unresolved issues such as impaired re-endothelialization and subsequent thrombosis induction [10]. On the other hand, the clinic use of strong PPARγ-activating drugs were either suspended or restricted because of their adverse effects including weight gain, liver injury and the increased risk of cancer, and cardiovascular events [11]. Such problems make the characterization of other compounds able to suppress VSMC migration highly clinically relevant. To this end, the implication of natural plant-derived compounds in controlling the migration of VSMCs in diseased arteries has been widely studied in the last decade. *Evodia rutaecarpa* (Chinese name: Wu-Chu-Yu) has been used to make spicy sauce for a long history, and is a traditional Chinese medicine used for the treatment of headache, hypertension, dyspepsia, gastrointestinal dysfunction, and so on. Evodiamine (Figure 1A) is the major ingredient isolated from the fruit of *E. rutaecarpa*. As a quinolone alkaloid, it possesses many biological effects, such as anti-inflammatory, anti-tumor, anti-obesity, vascular relaxing, thermoregulatory and uterine contracting effects [12]. Of note, evodiamine exhibits anti-tumor properties by inhibiting motility of various cancer cell lines. For exapmle, it has been reported that evodiamine suppresses migration of several tumor cell lines induced by hepatocyte growth factor partly through retardation of cell spreading [13]. In addition, evodiamine represses the migration and invasion of human breast cancer cells by downregulating the expression of MMP-9, urokinase-type plasminogen activator (uPA), and uPA receptor (uPAR) [14]. More interestingly, evodiamine shows a protective effect on the cardiovascular system. For example, in isolated rat mesenteric arteries, evodiamine exhibits a vasodilative effect [15]. Evodiamine also acts as a diuretic through inhibiting the releasing of aldosterone, which plays a role in blood volume controlling [16]. Moreover, evodiamine inhibits LIGHT-induced production of ROS and pro-inflammatory cytokines, the phosphorylation of MAPKs (p38 and ERKs), and the activation of NADPH oxidase in human monocytes [17]. These findings suggest that evodiamine has the potential to treat cardiovascular diseases.

Although evodiamine has been proven to retard the metastasis of tumor cells and is beneficial for the cardiovascular system, whether evodiamine possesses similar actions in VSMCs remains unknown. Therefore, in the present study, we sought to investigate the anti-migrative activity and the potential target of evodiamine in PDGF-BB-stimulated VSMCs.

## 2. Results

### 2.1. Evodiamine Inhibits Platelet-Derived Growth Factor (PDGF)-BB-Induced Vascular Smooth Muscle Cell (VSMC) Migration

We first assessed the cytotoxicity of evodiamine prior to investigating its effect on VSMC migration. The Cell Counting Kit-8 (CCK-8) assay results revealed that evodiamine at the concentrations lower than 2 μM did not inhibit cell viability over 30 h (Figure 1B). We therefore selected 0.1 and 0.5 μM of evodiamine as the safe doses for VSMCs and used them throughout our study. We then used a wound healing migration assay to evaluate the effect of evodiamine on PDGF-BB-induced VSMC migration. In contrast to the unchanged migration rate of VSMCs with the treatment of evodiamine alone, the wound closure rate reached to approximately a four-fold higher level upon stimulation with 10 ng/mL PDGF-BB for 24 h, compared to the untreated control. However, treatment with evodiamine at 0.1 or 0.5 μM inhibited PDGF-BB-induced VSMC migration in a dose-dependent manner (Figure 1C). The inhibitory effect of evodiamine on VSMC migration was further confirmed by a transwell migration assay. Figure 1D shows that the number of VSMCs able to move across the membrane was significantly reduced by evodiamine. Inhibition percentage of migrated cells treated with evodiamine (0.1 or 0.5 μM) was 40% and 60%, respectively, compared with the PDGF-BB-stimulated cells.

### 2.2. Evodiamine Inhibits the Expression of Migration-Associated Regulators

**Figure 2:**
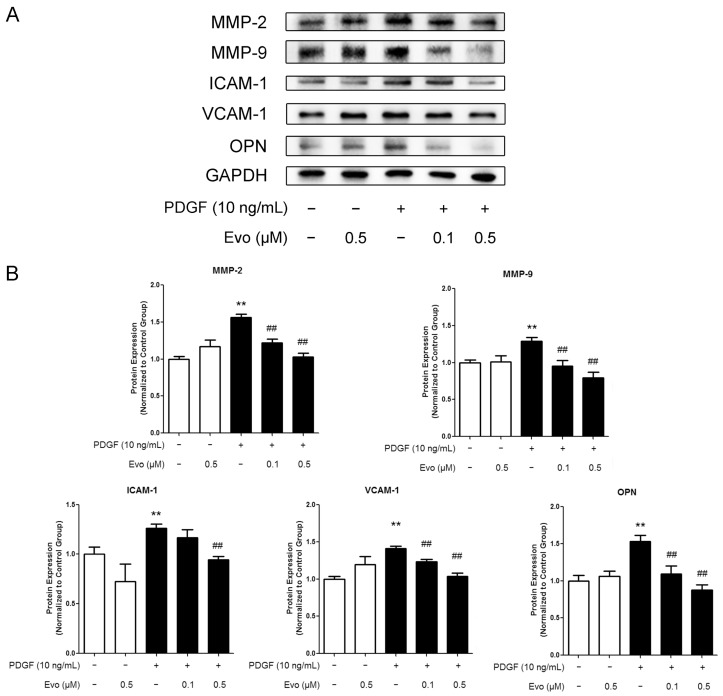

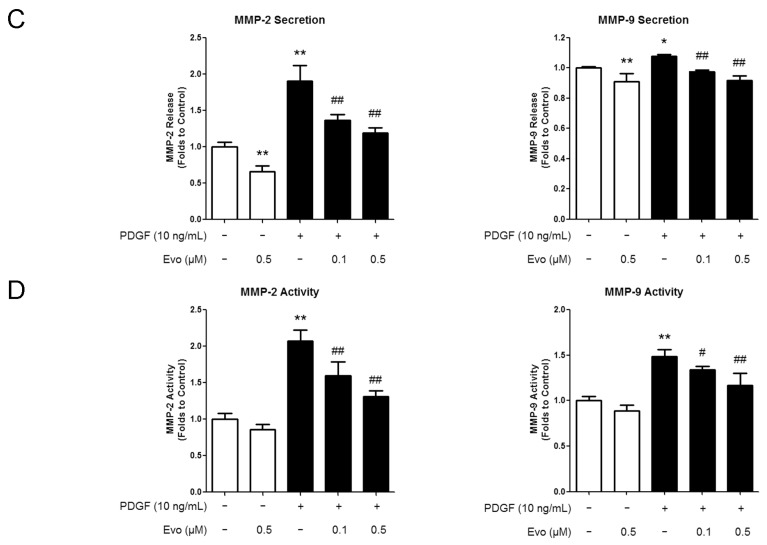
Evodiamine inhibits the expression of migration-associated regulators. (**A**) VSMCs were pretreated with evodiamine for 6 h and then stimulated with PDGF-BB for another 24 h. Western blot was used to measure the protein expression levels of regulators involved in the VSMC migration, including matrix metalloproteinase (MMP)-2, MMP-9, intercellular adhesion molecule-1 (ICAM-1), vascular cell adhesion molecule-1 (VCAM-1), and osteopontin (OPN). For both MMP-2 and MMP-9, their latent forms (72 and 92 kDa, respectively) were detected; (**B**) The signal ratio of examined protein to the internal control glyceraldehyde-3-phosphate dehydrogenase (GAPDH) was quantified by densitometric scanning; (**C**) The concentrations of MMP-2 and -9 in the culture medium were detected by Enzyme Linked Immunosorbent Assay (ELISA); (**D**) The activities of MMP-2 and MMP-9 were determined by using their respective Biotrak Activity Assay Systems. Evo, evodiamine. Data are presented as mean values ± SD of three independent experiments. * *p* < 0.05, ** *p* < 0.01 compared with the control group; ^#^ *p* < 0.05, ^##^ *p* < 0.01 compared with the PDGF-BB-stimulated group.

According to previous studies, the increased expression of MMPs induced by PDGF-BB plays an important role in facilitating ECM degradation and promoting VSMC motility, which is tightly linked to the development of atherosclerosis [18]. On the other hand, intercellular adhesion molecule-1 (ICAM-1), vascular cell adhesion molecule-1 (VCAM-1), and osteopontin (OPN) are inflammation-associated proteins, and are also key regulators involved in cell migration [19,20]. Western blot results showed that PDGF-BB treatment consistently increased the protein expression levels of all these factors in VSMCs (Figure 2A,B) and evodiamine was found to inhibit such increases in a dose-dependent manner. Enzyme Linked Immunosorbent Assays (ELISA) also indicated that evodiamine suppressed the concentrations of MMP-2 and MMP-9 released to the medium, which was originally upregulated by PDGF-BB. The extents of suppression were 40% and 16%, respectively, compared to the PDGF-BB-stimulated group (Figure 2C). We also used Biotrak Activity Assay Kits (GE Healthcare) to quantify actual enzymatic activities of MMP-2 and MMP-9 in their active form and our results indicated that evodiamine indeed suppressed PDGF-BB-induced upregulation in their activities (Figure 2D). These findings indicate that evodiamine inhibits PDGF-BB-induced VSMC migration through downregulating migration-related proteins.

### 2.3. Evodiamine Increases Peroxisome Proliferator-Activated Receptor γ (PPARγ) Expression and Nuclear Translocation in VSMCs

**Figure 3:**
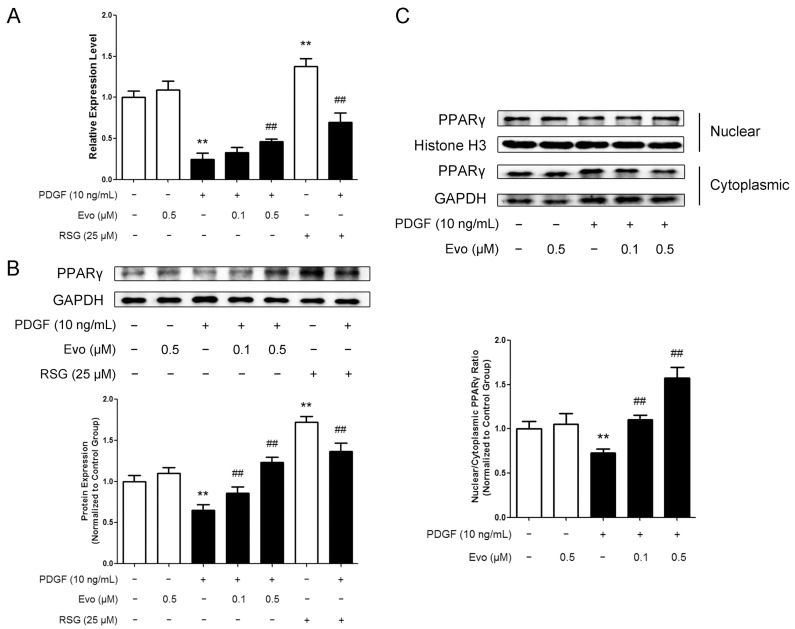
Evodiamine activates peroxisome proliferator-activated receptor γ (PPARγ) in PDGF-BB-stimulated VSMCs. (**A**) VSMCs were pretreated with evodiamine or rosiglitazone for 6 h and then stimulated with PDGF-BB for another 24 h. RT-qPCR was used to measure the mRNA expression levels of PPARγ; (**B**) Western blot was used to measure the protein levels of PPARγ. The signal ratio of PPARγ to the internal control GAPDH is shown at the bottom; (**C**) Nuclear and cytoplasmic proteins were extracted separately, and Western blot was used to measure the protein levels of PPARγ in both fractions. The ratio of nuclear/cytoplasmic PPARγ is shown at the bottom. Evo, evodiamine. RSG, rosiglitazone. Data are presented as mean values ± SD of three independent experiments. ** *p* < 0.01 compared with the control group; ^##^ *p* < 0.01 compared with the PDGF-BB-stimulated group.

Evodiamine has been proven to be a PPARγ activator [21,22]. Therefore, we examined whether the expression of PPARγ was also affected by evodiamine in VSMCs. We found that although rosiglitazone, a well-known PPARγ agonist, significantly induced PPARγ expression both at the mRNA and protein levels, evodiamine alone did not regulate PPARγ expression. In contrast, an obvious decrease was observed in VSMCs when treated with PDGF-BB, which was dose-dependently reversed by evodiamine (Figure 3A,B).

On the other hand, since the increased nuclear translocation from cytosol is an important characteristic of activated PPARγ, we also analyzed the distribution of PPARγ in the nucleus and cytoplasm of VSMCs. We found that PDGF-BB inhibited, while evodiamine promoted, the ratio of nuclear/cytoplasmic PPARγ (Figure 3C). All these findings suggest that PPARγ is one of the pharmaceutical targets of evodiamine.

### 2.4. PPARγ Mediates the Actions of Evodiamine in Regulating VSMC Migration

**Figure 4:**
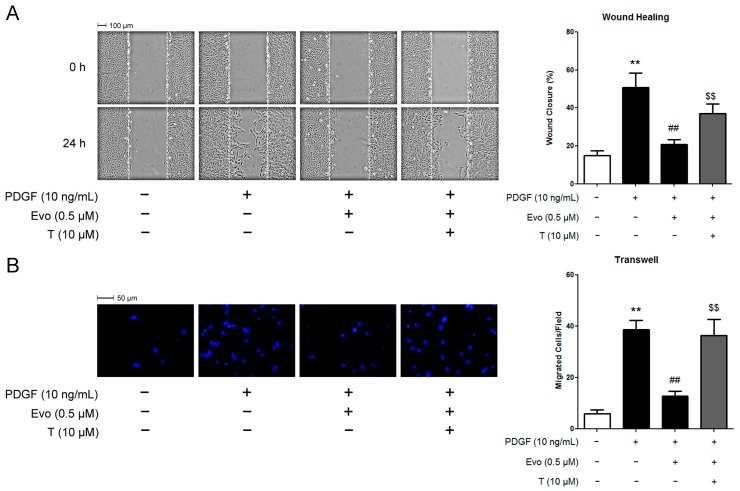
PPARγ antagonist alleviated the inhibitory effects of evodiamine on PDGF-BB-stimulated VSMC migration. VSMCs were pretreated with 0.5 μM evodiamine for 6 h and then stimulated with 10 ng/mL PDGF-BB for 24 h (wound healing) or 6 h (transwell). 10 μM T0070907 was given 0.5 h in advance of evodiamine treatment when required. Cell migration was measured by using a wound healing assay (**A**) or a Boyden chamber transwell assay (**B**). In Panel B, blue color indicates cell nuclei stained with DAPI. Evo, evodiamine. T, T0070907. Data are represented as mean values ± SD of three independently prepared samples each with five measurements. ** *p* < 0.01 compared with the control group; ^##^ *p* < 0.01 compared with the PDGF-BB-stimulated group; ^$$^ *p* < 0.01 compared with the group treated with both evodiamine and PDGF-BB.

Since evodiamine activates PPARγ and PPARγ has been shown to inhibit VSMC abnormal activation including excess proliferation and migration [6,23], we wonder whether the inhibitory effect of evodiamine on VSMC migration is PPARγ-dependent. To explore this possibility, we treated VSMCs with 10 μM T0070907, which is a PPARγ antagonist decreasing the phosphorylation of PPARγ and impairing its DNA-binding ability. As shown in Figure 4A, the wound healing assay results demonstrated that 0.5 μM of evodiamine retarded PDGF-BB-induced VSMC migration by 60%. Interestingly, pretreatment of cells with T0070907 partially released the block of VSMC migration by evodiamine. Results from the transwell assay showed a similar trend (Figure 4B).

**Figure 5:**
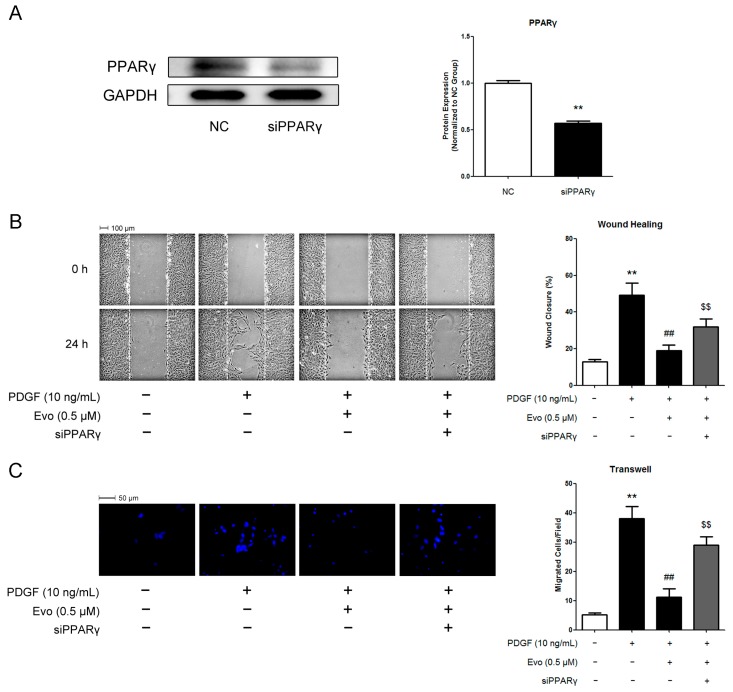
Knockdown of PPARγ by its specific siRNA alleviated the inhibitory effects of evodiamine on PDGF-BB-stimulated VSMC migration. (**A**) Knockdown efficiency of siRNA tested by Western blot; (**B**,**C**) Quantification of VSMC migration. VSMCs were transfected with scrambled siRNA (negative control, or NC) or siRNA against PPARγ (siPPARγ). 24 h after transfection, cells were pretreated with 0.5 μM evodiamine for 6 h and then stimulated with 10 ng/mL PDGF-BB for 24 h (wound healing assay, **B**) or 6 h (transwell assay, **C**). In Panel C, blue color indicates cell nuclei stained with DAPI. Evo, evodiamine. Data are represented as mean values ± SD of three independently prepared samples each with five measurements. ** *p* < 0.01 compared with the control group; ^##^ *p* < 0.01 compared with the PDGF-BB-stimulated group; ^$$^ *p* < 0.01 compared with the group treated with both evodiamine and PDGF-BB.

To exclude the possibility that the functions of T0070907 in VSMCs may be due to non-specific side-effects other than a blockade of PPARγ, we directly knocked down the expression of PPARγ in VSMCs by siRNA transfection. As shown in Figure 5A, specific siRNA oligonucleotides against PPARγ successfully suppressed the protein expression levels of PPARγ in VSMCs. In addition, both the wound healing assay and the transwell assay indicated that knockdown of PPARγ alleviated the inhibitory effects of evodiamine on PDGF-BB-stimulated VSMC migration (Figure 5B,C), which was coincided with the observations in VSMCs treated with T0070907.

## 3. Discussion

Evodiamine has been reported to have anti-inflammatory and anti-tumor properties, thus has attracted a considerable interest in its health effects. Several studies have also indicated that evodiamine has a protective effect on the cardiovascular system, although the possible mechanisms and potential molecular targets remain unknown [24]. Given that the dysregulated migration of VSMCs into the intima contributes importantly to the development of various cardiovascular diseases, the attenuation of accelerated VSMC migration could be one mechanism through which evodiamine exerts its beneficial actions. Indeed, in the present study, we demonstrated that evodiamine inhibited PDGF-BB-induced VSMC migration in a dose-dependent manner at the non-cytotoxic doses. Treatment with evodiamine suppressed the expressions of migration-related regulators such as MMPs, adhesion molecules and OPN. More importantly, we provided evidence to show that PPARγ is one of the major targets mediating the inhibitory effect of evodiamine on VSMC migration. To the best of our knowledge, this is the first time to show that evodiamine regulates VSMC pathophysiological processes.

The degradation of various components, such as the basement membrane and the elastic lamina, is essential for the migration of VSMCs through the matrix. The MMP family plays an essential role in this process. Pro-MMP-2 is constitutively expressed in VSMCs both *in vivo* and *in vitro*. However, the expression of other MMPs, such as MMP-9, is inducible. In atherosclerotic lesions and balloon injuries, their expression can be induced by cytokine stimulation [25]. Therefore, the downregulation of MMPs leads to inhibition of VSMC migration, and consequently ameliorates intimal hyperplasia in the rat vascular injury model [26]. Similarly, the ability of MMPs to degrade ECM proteins also regulates tumor behavior. Recent evidences indicate that rather than promoting tumor cell extravasation, MMPs are likely to induce intravasation, the process to penetrate the circulation following invasion of blood vessels [27]. In the present study, we showed that evodiamine inhibited the protein expression levels, secretion and activities of MMP-2 and -9 in PDGF-BB-stimulated VSMCs. Furthermore, the ability of evodiamine to inhibit the expression of MMPs has also been confirmed in human prostate and breast cancer cells [14,28]. These findings suggest that the inhibitory effect of evodiamine on MMP expression may be universal and provides the molecular basis for its ability to retard cell motility.

Inflammatory stimuli such as proinflammatory cytokines and free fatty acids have been considered as a critical contributor to VSMC migration. They also increase ROS production and cause oxidative stress. On the other hand, oxidative stress promotes the generation of inflammatory factors, such as cytokines, chemokines, and adhesion molecules. Moreover, oxidative stress activates the adhesion of lymphocytes. Therefore, inflammation and oxidative stress construct a self-perpetuating cycle, which contributes importantly to the pathogenesis of cardiovascular diseases [29]. In the present study, evodiamine was shown to decrease the expression of ICAM-1 and VCAM-1, which are representative adhesion molecules, and OPN, which is involved in the inflammation and migration of VSMCs. However, a recent study showed that evodiamine increased the expression of adhesion molecules in human gastric adeno-carcinoma (AGS) cells although it still had anti-proliferative and anti-migratory activities in these cells [30]. The contradictory results may be resulted from the virtual difference between the tumor cells and VSMCs.

As a versatile nuclear receptor, PPARγ is expressed in VSMCs of normal vascular wall and atherosclerotic lesions in humans. PPARγ is also present in rodent VSMCs and rat neointima induced by balloon injury of aortas [31]. The beneficial roles of PPARγ in regulating the homeostasis of cardiovascular system, particularly the inhibitory effect on VSMC migration, have been extensively studied. First, Ets-1 is a transcriptional factor mediating the expression of MMPs, and is induced by PDGF-BB in cultured VSMCs or by balloon injury in rat aortas. PPARγ activation by its agonists suppresses the protein expression of Ets-1, thus inhibits mRNA and protein expression, as well as enzymatic activity of MMP-9 [32]. In addition, as mentioned above, expression of adhesion molecules by vessel cells leads to adhesion of leukocytes, which is a critical early event in the development of atherosclerosis. PPARγ ligands are also able to inhibit the expression of ICAM-1, VCAM-1, and decrease the production of chemokines in VSMCs [33]. Thus, activation of PPARγ is an important strategy to control VSMC migration. Our findings demonstrated that evodiamine increased PPARγ expression and promoted its nuclear translocation only in PDGF-BB treated VSMCs, but not in untreated control cells. In contrast, rosiglitazone non-selectively activated PPARγ in both groups. These results are of particular interest and imply that evodiamine limits its regulation towards PPARγ only in abnormally activated VSMCs. Therefore, regulatory activity of evodiamine on PPARγ may be a secondary rather than primary effect under the presence of PDGF-BB. It has been shown that PDGF-BB activates several transcriptional factors and regulatory genes, which in turn represses PPARγ [34,35]. And evodiamine may antagonize the induction of these factors, thus restoring the expression and activity of PPARγ. For example, while PDGF-BB is able to activate β-catenin, a versatile protein regulating the intercellular adhesion and gene transcription, evodiamine has been reported to inhibit the expression of this factor through the Wnt signaling pathway in gastric cancer stem cells [34,36]. It is interesting to investigate that whether β-catenin mediates the restoration of PPARγ by evodiamine in PDGF-BB-stimulated VSMCs. More importantly, blockade of PPARγ activity by its antagonist T0070907 or knockdown of PPARγ expression by its specific siRNA attenuated the inhibitory effect of evodiamine on VSMC migration, strongly suggesting that PPARγ at least partially mediates the actions of evodiamine in VSMCs. Thus, evodiamine may be safer for the clinic use when considering that classic PPARγ agonists have serious side-effects and some of them have been withdrawn from the market or have restricted prescription [11]. Of note, the precise role of PPARγ in mediating evodiamine’s effects on VSMCs should be confirmed in the future study by using VSMCs isolated from PPARγ knockout mice and the mechanisms through which evodiamine induces PPARγ expression and activity should also be evaluated.

## 4. Experimental Section

### 4.1. Chemicals and Materials

Evodiamine (Cat. No. S2382) and T007097 (Cat. No. S2871) were purchased from Selleck (Houston, TX, USA), and were dissolved in DMSO for the later use. Rosiglitazone maleate (Cat. No. K0101) was purchased from Yuan-Ye Biotech Inc. (Shanghai, China), and also dissolved in DMSO. PDGF-BB (Cat. No. P4056) was bought from Sigma Aldrich (St. Louis, MO, USA), and dissolved in 4 mM hydrochloric acid containing 0.1% bovine serum albumin. Triton X-100 (Cat. No. T8200) was bought from Solarbio (Beijing, China). Coomassie Brilliant Blue R-250 was bought from Biosharp (Hefei, China). Antibodies against MMP-2 (Cat. No. sc-10736), MMP-9 (Cat. No. sc-6840), ICAM-1 (Cat. No. sc-1511), and VCAM-1 (Cat. No. sc-1504) were purchased from Santa Cruz Biotechnology (Dallas, TX, USA). Antibodies against OPN (Cat. No. ab11503) and PPARγ (Cat. No. ab191407) were purchased from Abcam (Cambridge, MA, USA). Antibody against GAPDH (Cat. No. KC-5G5) was purchased from Kang-Chen Biotech Inc. (Shanghai, China). Antibody against Histone H3 (Cat. No. 4499) was purchased from Cell Signaling (Danvers, MA, USA).

### 4.2. Cell Culture

Male Sprague–Dawley rats aged at three to four-weeks-old were used for VSMC isolation. VSMCs were isolated from their thoracic aortas as previously described [37]. The cells were cultured in 5% CO_2_ at 37 °C. Only the cells at passages 4–8 were used in this study. For function analysis, VSMCs were pre-treated with evodiamine in the serum-free medium and then stimulated with 10 ng/mL PDGF-BB. 10 μM T0070907 was given 0.5 h prior to evodiamine treatment when indicated.

### 4.3. Cell Viability Assay

CCK-8 toxicity assay was used for VSMC viability analysis. Briefly, 5 × 10^3^ VSMCs were seeded into each well of a 96-well plate, and cultured at 37 °C overnight for attachment. Then the cell were transferred into 100 μL medium containing 10% fetal bovine serum and evodiamine at indicated concentrations and incubated for 30 h. After that, 10 μL WST-8 reagent (Cat. No. E1CK-000208, EnoGene, Nanjing, China) was added into each well, mixed with the medium, and incubated at 37 °C for 2 h. Finally, a microplate reader was used to measure the absorbance at 450 nm.

### 4.4. Wound Healing Assay

A wound healing assay was used to analyze the VSMC migration rate. Briefly, 1.5 × 10^5^ VSMCs were seeded into each well of 6-well plates and grew to confluence. After 24-h serum deprivation, the cells were treated with 0, 0.1, or 0.5 μM evodiamine for 6 h. Then three parallel wounds with similar widths (<3 mm) were created in each well by using a sterile 200 μL pipette tip (set as 0 h), and PDGF-BB was added into the wells for stimulation. By using direct microscopic visualization, a reference point at the bottom was created in each field of the wound at 0 h, and wound closure rates were analyzed by photographing and measuring the remaining cell-free area in the identical field immediately after 24-h stimulation.

### 4.5. Boyden Chamber Assay

A Boyden chamber assay was also used for VSMC migration analysis. In brief, a 24-well modified Boyden chamber with polycarbonate membranes (8 μm pore-size, Cat. No. 3422, Corning Costar, Cambridge, MA, USA) was coated with MaxGelTM ECM (Cat. No. E0282, Sigma Aldrich). Serum-free DMEM (500 μL) containing PDGF-BB and evodiamine at indicated concentrations were added in the lower chambers. 100 μL serum-free DMEM containing 2.5 × 10^4^ VSMCs was seeded into each upper chamber, which was placed atop a lower chamber. Then the cells were allowed to migrate in the incubation at 37 °C for 6 h. After that, the non-migrated cells were wiped off with cotton swabs from the superior surface of the membranes. The migrated cells, which were located on the inferior surface of the membrane, were stained with 4′,6-diamidino-2-phenylindole (DAPI) after fixation using 4% paraformaldehyde. A fluorescence microscope (Nikon, Tokyo, Japan) was used for photographing, and 5 random fields were selected for each membrane. Finally, the number of cells in each field was counted for statistical analysis.

### 4.6. Western Blotting Analysis

Western blotting was executed as previously described [38]. For total protein extraction, VSMCs were lysed in RIPA lysis buffer. For the extraction of nuclear and cytoplasmic proteins, a nuclear/cytosol protein extraction kit (Cat. No. BSP001, Sangon, Shanghai, China) was used according to the manufacturer’s instructions. The protein concentrations were quantified using the Dc protein assay reagent (Cat. No. 5000116, Bio-Rad, Hercules, CA, USA). 20 μg of each protein was loaded on SDS-PAGE gel for electrophoresis, and transferred onto PVDF membrane (Cat. No. IPVH00010, Millipore, Bedford, MA, USA) after separation. The membranes were exposed to appropriate primary antibodies and then their corresponding secondary antibodies conjugated with horseradish peroxidase (Cat. No. sc-2005, sc-2004, and sc-2020, Santa Cruz Biotechnology) for visualization. The blots were quantified by densitometric scanning using the NIH Image J 1.32j software (Bethesda, MD, USA). GAPDH was used as the internal control. Three independent experiments were performed, and the signal ratios of examined proteins to GAPDH were analyzed and presented using histogram.

### 4.7. Matrix Metalloproteinase (MMP)-2 and MMP-9 Activity Assays

The culture supernatants of VSMCs were collected by centrifuging at 1000× *g* for 20 min, and concentrated 10× using Centriprep 10,000 MWCO tubes (Millipore) by centrifuging at 4900 rpm for 30 min. The levels of active MMP-2 and MMP-9 were measured by using MMP-2 and MMP-9 Biotrak Activity Assay Kits (Cat. No. RPN2631, and RPN2634, GE Healthcare, Piscataway, NJ, USA) according to the manufacturer’s protocol. The protein concentrations in the supernatants were quantified using the Dc protein assay reagent (Cat. No. 5000116, Bio-Rad, Hercules, CA, USA). The levels of active MMP-2 and MMP-9 in the culture supernatants were normalized to the total protein contents in each sample.

### 4.8. Enzyme Linked Immunosorbent Assay (ELISA)

The levels of secreted MMP-2 and MMP-9 in the culture supernatants were analyzed using commercial ELISA kits (Cat. No. SEA100Ra, and SEA553Ra, Cloud-Clone Corp., Houston, TX, USA) according to the manufacturer’s protocol.

### 4.9. Real-Time RT-PCR

Trizol reagent (Cat. No. 15596-026, Invitrogen, Carlsbad, CA, USA) was used for RNA extraction from VSMCs. 1 μg of total RNA and HiScript™ Q RT SuperMix for qPCR (Cat. No. R122-01, Vazyme, Nanjing, China) were used for the reverse transcription. For real-time PCR analysis, SYBR premix Ex Taq (Cat. No. Q111, Vazyme) was used for the quantification of mRNA expression levels of each gene, and amplification was executed using the LightCycler^®^ Nano System (Roche, Basel, Switzerland). The expression levels of mRNAs were normalized to that of 18S ribosomal RNA, which served as an internal control. Primer sequences were: PPARγ forward: 5’-CCCTGGCAAAGCATTTGTAT-3’, reverse: 5’-ACTGGCACCCTTGAAAAATG-3’; 18S forward: 5’-AAACGGCTACCACATCCAAG-3’, reverse: 5’-CCTCCAATGGATCCTCGTTA-3’. The amplification conditions were as follows: 95 °C for 10 min as the initial denaturation step, and 45 cycles of amplification consisting of 95 °C for 10 s, and 60 °C for 30 s. The melting curve of each pair of primers was analyzed prior to the formal experiments to guarantee the specificity. The threshold cycle (*C*_t_) values were calculated using the instrument’s software, and the 2^−ΔΔ*C*t^ method was used for the calculation of the relative expression levels, which were presented as folds to the average expression level of the respective gene in the control group. Three independent experiments were performed, and the relative expression levels were presented using histogram.

### 4.10. PPARγ RNAi

The rat PPARγ-specific siRNA (5’-GCUCCAAGAAUACCAAAGUTT-3’) and non-specific siRNA (Scrambled siRNA, 5’-UUCUCCGAACGUGUCACGUTT-3’) were designed and synthesized by GenePharma (Shanghai, China). To knockdown the expression of PPARγ in VSMCs, the cells were transfected with scrambled siRNA (NC) or siRNA against PPARγ (siPPARγ) 24 h in advance of indicated treatments. For knockdown efficiency test, proteins were collected 48 h after transfection of siRNAs. The transfection of siRNAs were performed using Lipofectamine 3000 reagent (Cat. No. L3000015, Invitrogen) according to the manufacturer’s instructions.

### 4.11. Data Analysis

Graphpad Prism 5 software (GraphPad Software Inc., San Diego, CA, USA) was used to analyze the data, which were presented as mean ± standard deviation (SD). Comparisons between two groups of data were executed with a one-way ANOVA analysis followed by Fisher’s LSD *post hoc* test. A value was considered statistically significant when *p* < 0.05.

## 5. Conclusions

In summary, evodiamine suppresses PDGF-BB-induced MMP and adhesion molecule expression, and consequent VSMC migration, by induction of PPARγ expression and nuclear translocation. These results indicate that evodiamine might possess a promising anti-atherogenic effect through attenuation of VSMC migration via activating PPARγ.
